# Molecular Cloning and Functional Characterization of a Novel (Iso)flavone 4′,7-*O-*diglucoside Glucosyltransferase from *Pueraria lobata*

**DOI:** 10.3389/fpls.2016.00387

**Published:** 2016-03-31

**Authors:** Xin Wang, Rongyan Fan, Jia Li, Changfu Li, Yansheng Zhang

**Affiliations:** CAS Key Laboratory of Plant Germplasm Enhancement and Specialty Agriculture, Wuhan Botanical Garden, Chinese Academy of SciencesWuhan, China

**Keywords:** glucosyltransferase, isoflavone, flavone, 4′, 7-*O-*di-glucosylation, 4′-*O-*glucosylation, *Pueraria lobata*

## Abstract

*Pueraria lobata* roots accumulate a rich source of isoflavonoid glycosides, including 7-*O-* and 4′-*O-*mono-glucosides, and 4′,7-*O-*diglucosides, which have numerous human health benefits. Although, isoflavonoid 7-*O-*glucosyltranferases (7-*O-*UGTs) have been well-characterized at molecular levels in legume plants, genes, or enzymes that are required for isoflavonoid 4′-*O-* and 4′,7-*O-*glucosylation have not been identified in *P. lobata* to date. Especially for the 4′,7-*O-*di-glucosylations, the genetic control for this tailing process has never been elucidated from any plant species. Through transcriptome mining, we describe here the identification and characterization of a novel UGT (designated PlUGT2) governing the isoflavonoid 4′,7-*O-*di-glucosylations in *P. lobata*. Biochemical roles of PlUGT2 were assessed by *in vitro* assays with PlUGT2 protein produced in *Escherichia coli* and analyzed for its qualitative substrate specificity. PlUGT2 was active with various (iso)flavonoid acceptors, catalyzing consecutive glucosylation activities at their *O-*4′ and *O*-7 positions. PlUGT2 was most active with genistein, a general isoflavone in legume plants. Real-time PCR analysis showed that PlUGT2 is preferentially transcribed in roots relative to other organs of *P. lobata*, which is coincident with the accumulation pattern of 4′-*O-*glucosides and 4′,7-*O-*diglucosides in *P. lobata*. The identification of PlUGT2 would help to decipher the *P. lobata* isoflavonoid glucosylations *in vivo* and may provide a useful enzyme catalyst for an efficient biotransformation of isoflavones or other natural products for food or pharmacological purposes.

## Introduction

**FIGURE 1:**
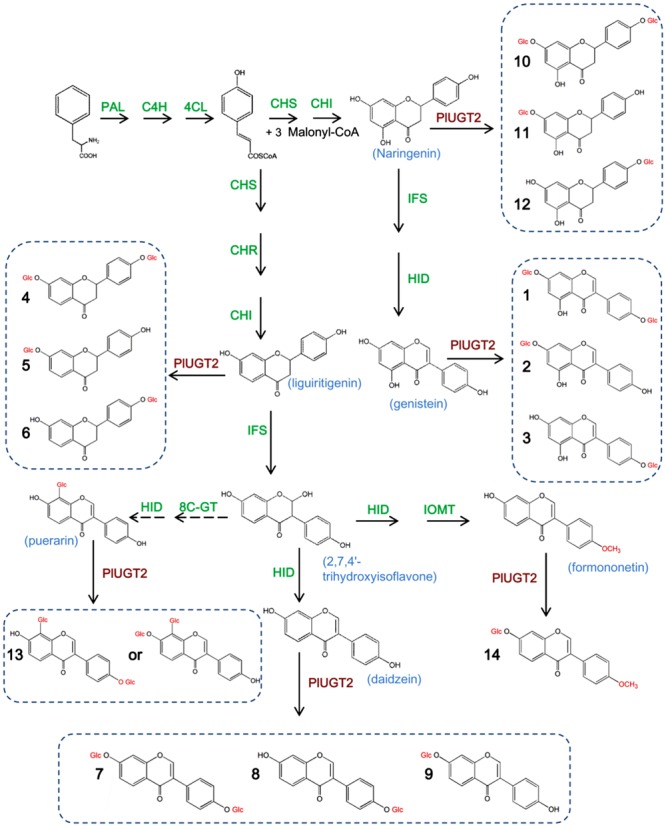
**Proposed pathways for isoflavone glucosylations catalyzed by PlUGT2 in *Pueraria lobata*.** PAL, phenylalanine ammonia-lyase; C4H, cinnamate 4-hydroxylase; 4CL, 4-coumarate:CoA ligase; CHS, chalcone synthase; CHI, chalcone isomerase; CHR, chalcone reductase; IFS, isoflavone synthase; HID, 2-hydroxyisoflavanone dehydratase; PlUGT2, *P. lobata* UDP-glucosyltransferase 2; IOMT, isoflavone *O-*methyltranferases. The dotted arrow indicates that the enzymes involved were not yet clear. (1) genistein 4′,7-*O-*diglucoside; (2) genistin (genistein 7-*O-*glucoside); (3) sophoricoside (genistein 4′-*O-*glucoside); (4) liquiritigenin 4′,7-*O-*diglucoside; (5) neoliquiritin (liquiritigenin 7-*O-*glucoside); (6) liquiritin (liquiritigenin 4′-*O-*glucoside); (7) daidzein 4′,7-*O-*diglucoside; (8) daidzin (daidzein 7-*O-*glucoside); (9) daidzein 4′-*O-*glucoside; (10) naringenin 4′,7*-O-*diglucoside; (11) naringenin 7*-O-*glucoside; (12) naringenin 4′*-O-*glucoside; (13) purearin mono*-*glucoside; (14) ononin (formononetin 7-*O*-glucoside).

The formation of isoflavonoid scaffold is conserved in legumes (Veitch, 2009; Ferreyra et al., 2012; Cheynier et al., 2013), however, the isoflavonoid composition and content are largely different between the species due to a variety of modifications on the compound backbone by specific modifying enzymes, therefore conferring their unique health benefits for human. Such enzymatic modifications, including hydroxylation, glycosylation, methylation, and acylation, change the physiological roles of these molecules by altering their chemical properties and accumulation sites within plants (Deavours et al., 2006; Saito et al., 2013; Xiao et al., 2014). As one of the major chemical elaborations, glycosylation is very popular in plant flavonoid metabolisms and transfers sugar moieties to their parent rings, producing a wide variety of glycosylated isoflavonoids (**Figure 1**). The property of this sugar conjugation, such as the position of conjugation, the number of glycosyl moiety, and the glycosidic structures, is crucial for the biological activity of the compound (Gachon et al., 2005; Yonekura-Sakakibara and Hanada, 2011). Glycosylation reactions are catalyzed by uridine diphosphate (UDP)-sugar glycosyltransferases (UGTs) and UGTs acting on plant natural chemicals usually belong to family 1 UGTs, which are characterized by a plant UGT signature, the plant secondary product glycosyltransferase consensus sequence (PSPG) motif (Vogt and Jones, 2000). In higher plants, UGTs exist as very large families, for example, over 150 different family 1 UGT genes were identified in *Medicago truncatula* (Modolo et al., 2007), and 117 putative family 1 UGT genes were found in *Pueraria lobata* (Wang et al., 2015). Although, more than 100 putative UGT genes were predicted from the *P. lobata* species, only three UGTs were functionally characterized in *P. lobata* (He et al., 2011; Li et al., 2014).

Puerarin, daidzein, and genistein have been identified to be pharmacologically active principles in *P. lobata* (Ohshima et al., 1988; Rong et al., 1998; Yasuda et al., 2005; Song et al., 2014), and their occurrences are associated with numerous human health benefits, e.g., preventing cardiovascular diseases (Wong et al., 2011), and suppressing oxidative damages induced by chronic ischemia (Zhang et al., 2015) and estrogen deficiency (Tang et al., 2012). Although these molecules exhibit important bioactivities, low water solubility is a serious drawback for their further practical applications for the food and pharmaceutical purposes. Glycol transformation is a powerful method to increase water solubility. For example, the water solubility of puerarin was increased up to 14–18 times when it is glucosylated (Li et al., 2004; Jiang et al., 2008). Glycosylated forms of the above principles were also detected in *P. lobata* tissues, suggesting the presence of various UGTs specific for glycosylating these compounds. *P. lobata* roots accumulate the 7-*O-*glucosides of genistein and daidzein (Kinjo et al., 1987; Fang et al., 2006a; Veitch, 2009), the 4′-*O-*glucosides of genistein, daidzein, and puerarin (Ohshima et al., 1988; Fang et al., 2006b; Shi et al., 2006), and the 4′,7-*O-*diglucoside of daidzein (Kinjo et al., 1987). UGTs of *P. lobata* which glucosylate the 7-*O-*position of genistein and daidzein have been molecularly characterized (Li et al., 2014). However, for the 4′-*O-* and 4′,7-*O-*glucosylation of isoflavonoids in *P. lobata*, enzymes or genes that are required for these chemical modifications remain unidentified; in particular, enzymes catalyzing the 4′,7-*O-*di-glucosylation have never been isolated from any plant species.

The present study reports a RNA sequencing-based molecular cloning and biochemical characterization of a novel *P. lobata* UGT (designated PlUGT2) that *O-*glucosylates isoflavonoids at either *O-*4′ or *O*-7 position. Especially, PlUGT2 shows a successive glucosylations toward the acceptors (daidzein and genistein) at both *O-*4′ and *O*-7 positions, producing their corresponding 4′,7-*O-*diglucosides. The consistency of the substrate specificity, gene expression, and metabolite profiling suggests the proposed roles of PlUGT2 in *P. lobata*. The identification of PlUGT2 would help to decipher the *P. lobata* isoflavonoid tailoring process and affords the possibility of increasing water solubility to make relevant compounds suitably for food and clinical applications.

## Materials and Methods

### Plant Material and Chemicals

*Pueraria lobata* materials (roots, leaves, and stems) were collected from wildly grown *P. lobata* plants from the Wuhan Botanical Garden, Chinese Academy of Sciences. All plant materials were stored at -80°C for future use. Liquiritin and neoliquiritin were purchased from PureOne Biotechnology Company (Shanghai, China). Other isoflavonoid and flavonoid acceptor substrates were all purchased from Shanghai Source Leaf Biological Technology Company (Shanghai, China). All organic solutions used for high-performance liquid chromatography (HPLC) were from the Wuhan Analytical Reagent Company (Wuhan, China).

### Isoflavonoid Extraction from *P. lobata* Tissues

About one gram of plant materials (roots, leaves, and stems) were ground to a fine power in a mortar with liquid nitrogen which were then dried at 50°C for 48 h. 20 mg of dried plant materials were extracted with 2 ml of methanol for three times. The crude extracts were centrifuged at 6,000 rpm for 10 min, and supernatants were concentrated to dryness using a rotary evaporator at 40°C. The methanol extracts were then resolved in 1 ml of HPLC-grade methanol, and filtered through a 0.22-μm nylon syringe filter prior to HPLC and liquid chromatography–mass spectrometry (LC–MS) analysis.

### Selection of PlUGT Candidates

Twenty-two family 1 UGTs with a higher gene expression in *P. lobata* roots relative to its leaves were previously identified by RNA-sequencing technology (Wang et al., 2015). The deduced amino acid sequences of these 22 UGTs were aligned with other identified plant UGT members by the Clustal W algorithm. A phylogenetic tree was constructed by means of the neighbor-joining method (with 1000 bootstrap replications), using MEGA 6.0 program. Based on the results from the phylogenetic tree analysis, a UGT candidate, designated PlUGT2 (official UGT designation UGT88E20), was selected for the current study.

### Cloning and Heterologous Expression of PlUGT2

Using *P. lobata* root cDNA as the template, the open reading frame (ORF) of *PlUGT2* was amplified by reverse transcription PCR (RT-PCR) with gene specific primers (PlUGT2-F and PlUGT2-R, Supplementary Table S1). The amplified product was then gel-purified, digested with BamHI and EcoRI, and inserted into pGEX-2T (GE Healthcare) with the *PlUGT2* ORF fused with a glutathione *S*-transferase (GST) tag, yielding the construct pGEM-2T-PlUGT2. The construct were transferred into *Escherichia coli* (BL21) cells for recombinant protein expression. Single colony of the transgenic *E. coli* strain was inoculated in 300 ml LB medium containing 50 μg ml^-1^ of ampicillin. Bacteria was grown at 37°C to OD600 value of 0.4–0.6, the recombinant protein expression was induced by addition of 0.5 mM isopropyl-β-D-thiogalactopyranoside (IPTG) and the cells were incubated at 16°C for 16 h. After the culturing, the cells were pelleted by centrifugation, suspended in 50 mM Tris-HCl buffer (pH 8.0) and disrupted using a sonicator. The cell lysate was centrifuged and the soluble supernatant was then used for the further protein purification. The recombinant PlUGT2 was purified using Glutathione Sepharose 4B kit (GE Healthcare) according to the protocol provided, and desalted into enzyme assay buffer by a 30 kDa-cut off centrifugal filter (Millipore). The purity of the recombinant PlUGT2 was checked by an electrophoresis on 12% SDS-PAGE, its concentration was measured by use of Bradford assays.

### Enzyme Assay

Enzyme assays were performed in 200 μl of the reaction mixture, containing 50 mM Tris-HCl (PH 8.0), 5 mM UDP-glucose, 10 μg of the purified recombinant PlUGT2, 100–250 μM (iso)flavonoid acceptors. After 10–60 min of incubation at 30°C, the reactions were stopped with 200 μl of methanol, and 10 μl of the reaction products were directly applied for HPLC analysis. The concentration of substrates and incubation time for each substrate were given in Supplementary Table S1. The reaction mixture without the addition of the purified PlUGT2 was set as a control.

### HPLC and LC–MS Analysis

The reaction products were analyzed by an LC-20AT HPLC system (Shimadzu, Kyoto, Japan), using an Inertsil ODS-SP reverse phase column (250 mm × 4.6 mm, 5 μm, Shimadzu) at 25°C. Solvent A was 0.1% formic acid in Milli-Q water, and solvent B was HPLC-grade acetonitrile. The system was equilibrated at 14% B for 10 min, and samples were separated on the column at a flow rate of 0.8 ml/min using a water-acetonitrile gradient in the mobile phase (14–50% B for 35 min, 50–70% B for 2 min, and 70–14% B for 1 min). The assays were monitored at 260 nm for detection of isoflavones and their glucosides, and 280 nm for flavones and their respective glucosides.

Liquid chromatography–mass spectrometry analysis was performed on an Accela LC system coupled with TSQ Quantum Access Max mass spectrometer (Thermo Scientific, USA). The column and analysis method were same with the HPLC analyses as described above. The MS data were recorded with ranges of *m/z* 100–800. Other parameters were set according to the previous report (Li et al., 2014).

### Quantitative Real-Time Reverse Transcription PCR (qRT-PCR)

Total RNA from each tissues were extracted by use of EASYspin plant RNA extraction kit according to manufacturer’s instructions (Aidlab Biotechlogies, Co., Ltd, China). After removing the residual genomic DNA by RNase-free DNase I (Thermo, USA), first-strand cDNA was synthesized by MMLV reverse-transcriptase (Thermo, USA). The qRT-PCRs were performed in three biological replicates using a FastStart Universal SYBR Green Master Mix (Roche, Mannheim, Germany). The thermal cycling conditions were set as follows: 95°C for 10 min, followed by 40 cycles of 95°C for 15 s and then 60°C for 1 min. The transcript abundances were calculated using the comparative threshold cycle method. *P. lobata* Actin (GenBank accession no. HO708075) was used as an internal reference gene to normalize the variation of the cDNA templates. Specific primers (qPlUGT2-F and qPlUGT2-R) used for the qRT-PCR were shown in Supplementary Table S2.

## Results

### *P. lobata* Isoflavone Glucosides Mostly Accumulate in its Roots

**FIGURE 2:**
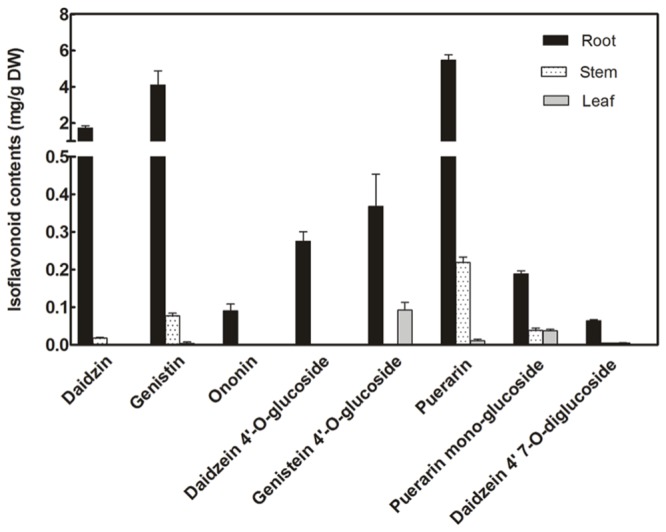
**Concentrations of isoflavone glucosides biosynthesized in different organs of *P. lobata***.

Isoflavonoid glucosides in *P. lobata* were analyzed by LC–MS analysis (**Figure 2**). The identity of these compounds was determined based on their HPLC retention times and individual mass spectrums in comparisons with corresponding authentic standards. The isoflavone glucosides detected here can be classified into four groups based on their glucosyl positions: 7-*O-*glucosides (daidzin, genistin, and ononin), 4′-*O-*glucosides (daidzein 4′-*O-*glucoside, genistein 4′-*O-*glucoside), 4′,7-*O-*diglucoside (daidzein 4′,7-*O-*diglucoside), and 8-*C-*glucosides (daidzein 8-*C-*glucoside, puerarin). For all these glucosides, roots are their main accumulation sites in *P. lobata*, especially, ononin and daidzein 4′-*O-*diglucoside were only found in the roots but not in the leaves and stems. Among these glucosides within *P. lobata* roots, 7-*O-*glucosides (daidzin and genistin) and 8-*C-*glucoside (puerarin) are predominant (daidzin, 1.72 ± 0.13 mg/g; genistin, 4.09 ± 0.78 mg/g; puerarin, 5.47 ± 0.29 mg/g) while 4′-*O-*glucosides and 4′,7-*O-*diglucoside accumulate at much lower amounts (**Figure 2**).

### Identification and Cloning of Full-Length cDNAs Encoding 4′-O-PlUGT

**FIGURE 3:**
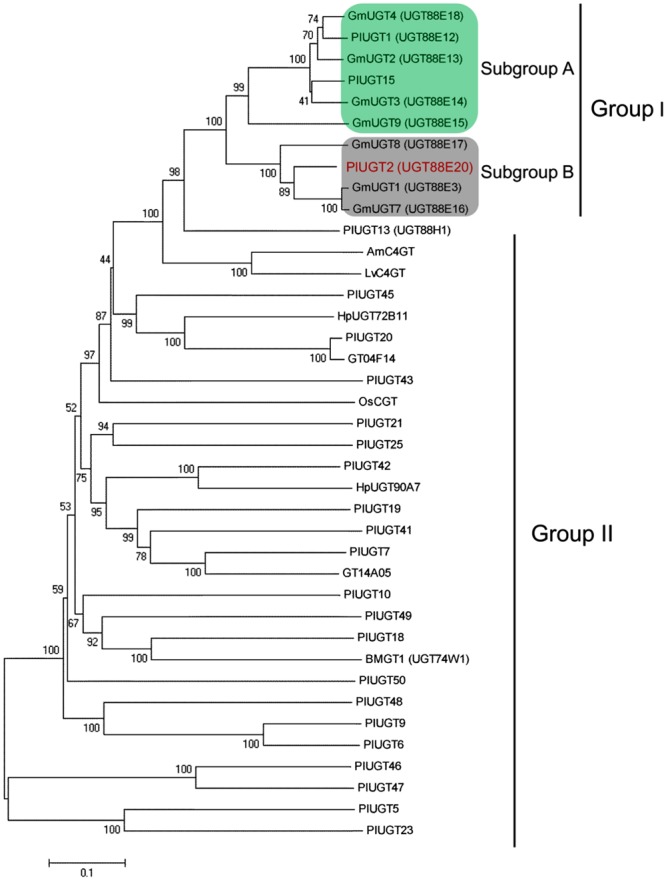
**Phylogenetic tree analysis of PlUGT2 with other known UGTs.** Twenty two identified *P. lobata* family 1 UGTs were aligned with other plant UGTs whose functions have been characterized, including seven soybean UGTs and three kudzu UGTs. The tree was constructed from a MEGA 6.0 program using a neighbor-joining method (with 1000 bootstrap replications). Names and accession numbers of UGTs used for the alignment are as follows. AmC4’GT (*Antirrhinum majus* UDP-glucose: chalcone 4′-*O-*glucosyltransferase, AB198665); BMGT1 (*Bacopa monnieri* genistein 4′-*O-*glucosyltransferase, UGT74W1, ACM09993); GmUGT2 (*Glycine max* UGT2, AB904891); GmUGT3 (*G. max* UGT3, AB904892); GmUGT4 (*G. max* UGT4, AB904893); GmUGT7 (*G. max* UGT7, AB904894); GmUGT8 (*G. max* UGT8, AB904895); GmUGT9 (*G. max* UGT9, AB904896); HpUGT90A7 (*Pilosella officinarum* flavonoid glucosyltransferases, ACB56926); HpUGT72B11 (*P. officinarum* coniferyl-alcohol glucosyltransferase, ACB56923); LvC4′GT (*Linaria vulgaris* UDP-glucose: chalcone 4′-*O-*glucosyltransferase, BAE48240); OsCGT (*Oryza sativa* flavoniod *C*-glucosyltransferase, CAQ77160). PlUGT1 (*P. lobata* isoflavone 7-*O-*glucosyltransferase 1, KC473565); PlUGT2 (*P. lobata* isoflavone 4′,7-*O-*glucosyltransferase, KU311040); PlUGT13 (*P. lobata* isoflavone 7-*O-*glucosyltransferase 13, KC473566); PlUGT15 (*P. lobata* glucosyltransferase 15, KU311041); PlUGT18 (*P. lobata* glucosyltransferase 18, KC473567); GT04F14 (*P. lobata* isoflavone 7-*O-*glucosyltransferase, HQ219042), GT14A05 (*P. lobata* flavone glucosyltransferase, HQ219047). The sequences of the other 20 *P. lobata* glucosyltransferases were submitted to the GenBank database with the accession nos. KU317800–KU317819.

By the use of RNA-sequencing technology, we previously reported 22 *P. lobata* family 1 *PlUGTs* that are preferentially expressed in its roots over leaves (Wang et al., 2015). For the isolation of 4′-*O-*UGTs from *P. lobata*, these 22 UGTs were phylogenetically analyzed with other plant UGTs whose functions have been characterized, which include seven soybean UGTs (Funaki et al., 2015) and three kudzu UGTs (PlUGT1, PlUGT13, and GT04F14; He et al., 2011; Li et al., 2014). The result showed that two putative PlUGTs (named as PlUGT2 and PlUGT15) were clustered into the same group with the soybean GmUGTs and PlUGT1 (group I), while the remaining 20 PlUGTs formed another group (group II; **Figure 3**). Furthermore, PlUGT2 and PlUGT15 were found to be scattered into two separate clades in the group I. PlUGT15 together with PlUGT1 displayed relatively higher homology to GmUGTs of subgroup A, whereas PlUGT2 showed a closer relationship with subgroup B members (Funaki et al., 2015). In subgroup A, GmUGT members (GmUGT3, GmUGT4, and GmUGT9) and PlUGT1 were characterized to be isoflavone specific 7-*O-*UGTs. The deduced amino acid sequence of PlUGT15 shared above 90% sequence identity with PlUGT1, and it indeed showed the same activity as PlUGT1 (data not shown). In subgroup B, PlUGT2 was in the same branch with GmUGT1 and GmUGT7, and had 82% amino acid sequence similarity to GmUGT1. It was reported that GmUGT1 and GmUGT7 not only efficiently glycosylated isoflavone aglycones at the 7-hydroxy group, but also exhibited considerable 4′-*O-*glucosylation activities toward some flavonoids. On the other hand, in the group II, PlUGT18 clustered with BMGT1 (UGT74W1) which specifically glucosylates the 4′-*O-*position of genistein (Ruby et al., 2014). The biochemical function of PlUGT18 was examined by our previous research and no activities toward any of the isoflavones were found (Li et al., 2014). Thus, taken together, the phylogenic tree analysis here made PlUGT2 an interesting candidate for functional characterization and testing of a possible role in the 4′-*O-*glucosylations of *P. lobata* isoflavonoids.

The full-length cDNA sequence of *PlUGT2* contained an ORF of 1,419 bp, which was predicted to encode a 463 amino acids UGT protein and officially assigned as UGT88E20. In multiple alignments, the deduced amino acid sequence of PlUGT2 showed a high degree of similarity to other representative UGTs from soybean and *P. lobata* (Supplementary Figure S1). It also contained a PSPG motif in the C-terminal region, which has been proposed to be the sugar donor binding site (Vogt and Jones, 2000).

### PlUGT2 is an Isoflavone-Specifically Bifunctional UGT

For *in vitro* biochemical assays, the recombinant PlUGT2 protein fused with a GST-tag was produced in *E. coli*. SDS-PAGE analysis of the soluble proteins from IPTG-induced *E. coli* cells expressing PlUGT2 showed a strong expression of recombinant PlUGT2 protein (Supplementary Figure S2). The molecular mass of purified protein was approximately 77 kDa, which was in agreement with the theoretically predicted molecular mass of PlUGT2 fused with the GST-tag.

**FIGURE 4:**
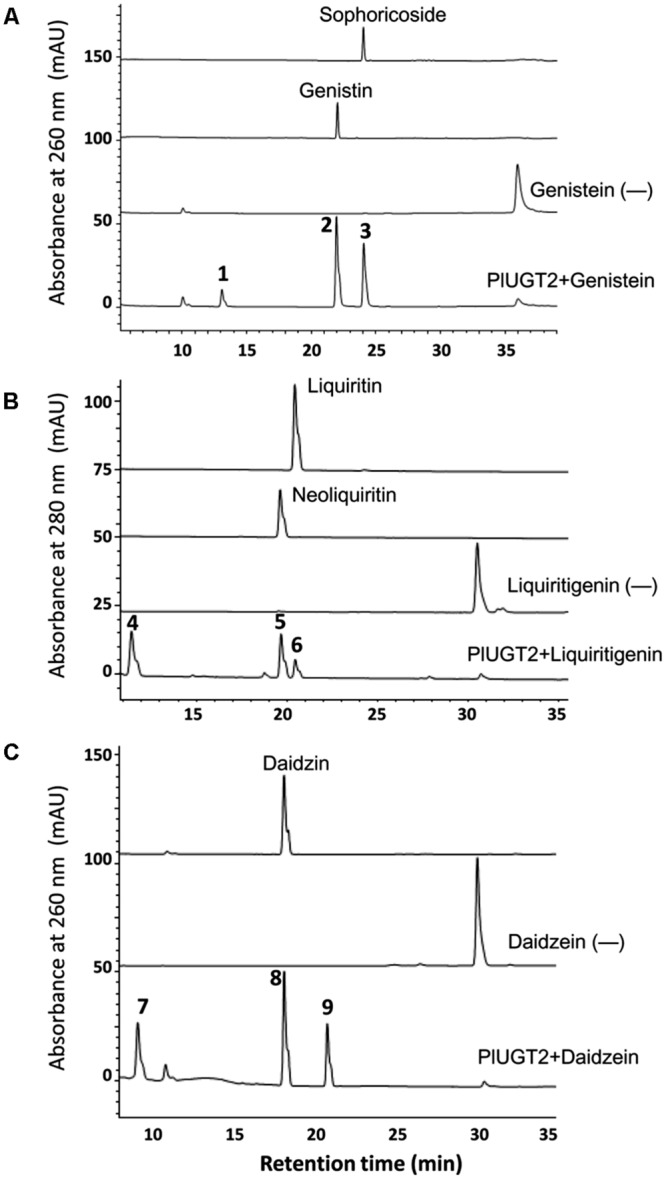
**High-performance liquid chromatography (HPLC) analysis of the products from the *in vitro* reactions of the recombinant PlUGT2 with genitein **(A)**, liquiritigenin **(B)**, and daidzein **(C)**.** PlUGT2 was able to converted these (iso)flavone aglycones to yield their respective 4′,7-*O-*diglucosides (peaks 1, 4, 7), 7-*O-*mono-glucosides (peaks 2, 5, 8), and 4′-*O-*mono-glucosides (peaks 3, 6, 9). (-) Indicates control reactions without the addition of PlUGT2. Peak 1, genitein 4′,7-*O-*diglucoside; peak 2, genitin (genitein 7-*O-*glucoside); peak 3, sophoricoside (genitein 4′-*O-*glucoside); peak 4, liquiritigenin 4′,7-*O-*diglucoside; peak 5, neoliquiritin (liquiritigenin 7-*O-*glucoside); peak 6, liquiritin (liquiritigenin 4′-*O-*glucoside); peak 7, daidzein 4′,7-*O-*diglucoside; peak 8, daidzin (daidzein 7-*O-*glucoside); peak 9, daidzein 4′-*O-*glucoside. The mass spectra of all the reaction products (peaks 1–9) were shown in Supplementary Figure S3, and their chemical structures are listed in the **Figure 1**.

In the *in vitro* enzyme assays, PlUGT2 was tested against various substrates and found to be active with some (iso)flavonoids using UDP-glucose as the sugar donor (**Table 1**). Among the active substrates, genistein was the best acceptor (100% relative activity, 136.96 ± 4.24 nmol mg protein^-1^ min^-1^) followed by formononetin (83.48% relative activity, 114.33 ± 4.35 nmol mg protein^-1^ min^-1^), daidzein (38.93% relative activity, 53.32 ± 1.95 nmol mg protein^-1^ min^-1^), liquiritigenin (11.83% relative activity, 16.20 ± 3.72 nmol mg protein^-1^ min^-1^), and naringenin (5.59% relative activity, 7.66 ± 1.32 nmol mg protein^-1^ min^-1^). Other (iso)flavonoids were poor substrates of PlUGT2 with their relative activities varying from 0.08 to 1.91% (**Table 1**). When genistein was used as the substrate, three new products (peaks 1–3) were formed in comparison with the control reaction (**Figure 4A**). By examining the retention times and mass spectra with chemical standards, the peak 2 and peak 3 could be identified as genistin (genistein 7-*O-*glucoside) and genistein 4′-*O-*glucoside (namely sophoricoside), respectively (Supplementary Figures S3A,B,G,H). This data suggested that PlUGT2 catalyzes either 7-*O-* or 4′-*O-*glucosylation activity toward genistein. The peak 2 and peak 3 had a molecular ion of *m/z^+^* 433 while the molecular ion for the peak 1 was 595 (Supplementary Figure S3F), indicating that the peak 1 could be genistein diglucoside in which a glucose group might be attached to both *O-*4′ and *O*-7 positions. To test this hypothesis, we attempted *in vitro* assays using genistin (genistein 7-*O-*glucoside) and sophoricoside (genistein 4′-*O-*glucoside) as the substrates. Apparently, the product peak 1 could be detected in both reactions (Supplementary Figures S4A,B), confirming that the peak 1 is genistein 4′,7-*O-*diglucoside. Thus, this suggested that PlUGT2 could transfer a glucose group to genistein at *O-*4′ or *O*-7 position and further convert the mono-glucosides to genistein diglucoside. It should be mentioned that the specific activity of PlUGT2 toward genistein mono-glucosides (0.15 ± 0.07 nmol mg protein^-1^ min^-1^ for genistin, 2.62 ± 0.16 nmol mg protein^-1^ min^-1^ for sophoricoside) was much lower than that toward its aglycone (136.96 ± 4.24 nmol mg protein^-1^ min^-1^; **Table 1**). Similarly, PlUGT2 transferred a glucose group to liquiritigenin at *O*-7 position to yield neoliquiritin (peak 5), at *O-*4′ position to yield liquiritin (peak 6), and both *O-*4′ and *O*-7 positions to produce the 4′,7-*O-*diglucoside of liquiritigenin (peak 4; **Figure 4B**). The peak 4 was postulated as the 4′,7-*O-*diglucoside of liquiritigenin based on its mass spectrum (Supplementary Figure S3I) and the results from the *in vitro* assays of PlUGT2 with neoliquiritin or liquiritin (Supplementary Figure S4C). The identities of the peak 5 and peak 6 were determined by comparing retention times and mass spectrums with their corresponding authentic chemicals (**Figure 4B**, Supplementary Figures S3C,D,J,K). The similar activity was also found in the reactions of PlUGT2 with daidzein (**Figure 4C**) and naringenin (Supplementary Figure S4D), forming the reaction product peaks 7–12. Except for the peak 8 (Supplementary Figures S3E,M), the authentic chemicals corresponding to the other peaks were not available. However, based on their mass spectrums (Supplementary Figures 3L,N,P–R) and the feature that the earliest eluted glucosides on the HPLC condition of this study were 4′,7-*O-*diglucosides which were successively followed by 7-*O-*glucosides and 4′-*O-*glucosides (**Figure 4**), the peaks 7–12 were postulated as follows: daidzein 4′,7-*O-*diglucoside (peak 7), daidzein 7-*O-*glucoside (peak 8), daidzein 4′-*O-*glucoside (peak 9), naringenin 4′,7-*O-*diglucoside (peak 10), naringenin 7-*O-*glucoside (peak 11), and naringenin 4′-*O-*glucoside (peak 12). When puerarin and formononetin were used as the substrates, only single product was obtained for each substrate. A single product (peak 13) was formed in the reaction with puerarin (Supplementary Figure S4E). The mass spectrum of the peak 13 indicated that there was a glucose group attached to puerarin (Supplementary Figure S3S). Since there are free hydroxyl groups at the *O-*4′ and *O*-7 positions of puerarin molecular, the peak 13 could be either puerarin 7-*O-*glucoside or puerarin 4′-*O-*glucoside (Shi et al., 2006). PlUGT2 converted the substrate formononetin to form a single product peak 14 which showed the same retention times and mass spectra with ononin (Supplementary Figures S3T and 4F), suggesting its 7-*O-*glucosylation activity toward formononetin.

**Table 1:** Enzyme activity of recombinant PlUGT2.

	Specific activities^a^	Relative
	(nmol mg protein^-1^ mi^-1^)	activity (%)^b^
4′*-O-*glucosylation activity		
Genistin	0.15 ± 0.07	0.11 ± 0.05
Daidzin	0.49 ± 0.06	0.36 ± 0.05
Purearin	0.19 ± 0.07	0.14 ± 0.05
Neoliquiritin^c^	0.87 ± 0.21	0.63 ± 0.15
7*-O-*glucosylation activity		
Sophoricoside	2.62 ± 0.16	1.91 ± 0.11
Formononetin	114.33 ± 4.35	83.48 ± 3.17
Liquiritin^c^	0.12 ± 0.01	0.08 ± 0.01
4′ 7-*O-*diglucosylation activity		
Genistein	136.96 ± 4.24	100.00 ± 3.09
Daidzein	53.32 ± 1.95	38.93 ± 1.43
Naringenin^c^	7.66 ± 1.32	5.59 ± 0.97
Liquiritigenin^c^	16.20 ± 3.72	11.83 ± 2.71
3′,4′,7-*O-*tri-glycusylation activity		
3′,4′,7-trihydroxyisoflavone	29.80 ± 2.94	21.76 ± 2.15

*^a^The specific activities were measured based on the initial velocity, and the values were calculated by the average of three replicates of the reactions. ^b^The relative activity toward genistein was set as 100, and that toward other substrates was normalized accordingly. ^c^Indicates that the acceptors are flavanones.*

To investigate whether PlUGT2 was able to glucosylate isoflavone aglycones at other hydroxyl group positions, 3′,4′,7-trihydroxyisoflavone, which has free hydroxyl groups at *O*-3′, *O*-4′, and *O*-7 positions, was used as the sugar acceptor for this enzyme. As a result, PlUGT2 showed a high specific activity toward 3′,4′,7-trihydroxyisoflavone (29.80 ± 2.94 nmol mg protein^-1^ min^-1^), which was comparable to that toward daidzein, yielding four additional peaks (peaks 15–18; Supplementary Figure S4G). The mass spectrums of these products indicated that the peaks 16–18 are mono-glucosides of 3′,4′,7-trihydroxyisoflavone while the peak 15 is a diglucoside of the substrate (Supplementary Figures S3U–X). The presence of these mono-glucosides demonstrated that PlUGT2 showed a 3′-*O*-, 4′-*O-*, or 7-*O-*glucosylation activity toward the acceptor. The chemical standards for the peaks 15–18 are not commercially available, but we speculated that the peak 15 might be the 4′,7-diglucoside of the substrate 3′,4′,7-trihydroxyisoflavone based on the activity feature of PlUGT2 described above.

### Tissue-Specific Expression Pattern of PlUGT2

**FIGURE 5:**
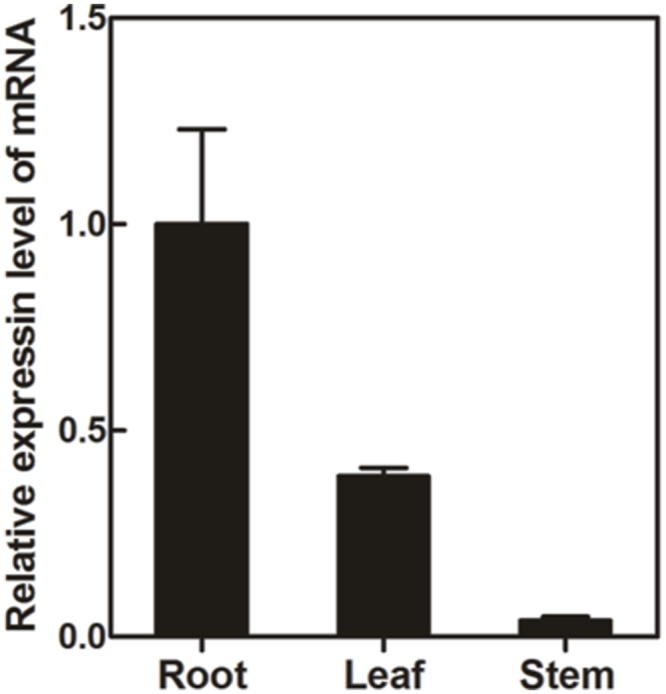
**Quantitative real-time reverse transcription PCR (qRT-PCR) analysis of the *PlUGT2* trancript abundance in different organs of *P. lobata*.** The transcript abundance of *PlUGT2* in different organs were normalized to the actin (internal reference) and expressed relative to the values of roots (control), which was set the value of 1. The data were derived from three biological replicates. The primer sequences used for qRT-PCR analysis are shown in Supplementary Table S2.

Transcript abundance of *PlUGT2* between *P. lobata* organs was compared by real-time PCRs. As shown in **Figure 5**, *PlUGT2* transcript was found in the roots, stems, and leaves of *P. lobata* with the highest expression being detected in roots. The transcript abundance of *PlUGT2* in the roots was 2.5- and 25-fold higher than that in the leaves and stems, respectively. Patterns of high *PlUGT2* transcrpts in *P. lobata* roots while relatively lower levels in its leaves and stems generally correlate with the accumulation pattern of the 4′-*O-* and 4′,7-*O-*isoflavone glucosides in *P. lobata* (**Figure 2**).

## Discussion

*P. lobata* roots are found to be useful in the treatment of diabetes, hyperlipidemia, and cardiovascular diseases (Wong et al., 2011, 2015). Isoflavonoids, including puerarin, daidzein, and genistein, are believed to be major bioactive components to contribute to the pharmacological actions (Keung et al., 1996; Prasain et al., 2007; Kayano et al., 2012). However, these isoflavonoids are very hydrophobic and their bioavailability is pretty low, therefore limiting their further applications for clinical trials. Glycol transformation is an effective method to improve water solubility of small molecular compounds, and the process is usually catalyzed by UGTs (Hansen et al., 2012). It has been reported that the difference in the positions of glycol-conjugations would affect their bioavailability and pharmacological properties (Gachon et al., 2005; Cheynier et al., 2013). The gluco-conjugations toward *P. lobata* isoflavonoids usually occur at either *O*-4′ or *O*-7, or at both positions. For example, mono-glucosides including 7-*O-*glucosides and 4′-*O-*glucosides, and 4′, 7-*O-*diglucosides have been detected in its roots (Du et al., 2011; Zhang et al., 2013). We previously reported the isolation of a *P. lobata* 7-*O-*UGT (designated PlUGT1) that could specifically attach a glucose group to daidzein or genistein at *O*-7 position (Li et al., 2014). In the present study, we aimed to isolate the genes encoding 4′-*O-*UGT and 4′,7-*O-*UGT from *P. lobata*.

A total of 117 unigenes encoding putative UGTs were identified in our previously constructed *P. lobata* transcriptome, in which 22 family 1 UGT genes are in full-length and show relatively higher expression levels in *P. lobata* roots relative to its leaves (Wang et al., 2015). Phylogenetic tree analysis of these 22 UGTs with some previously published UGTs showed that PlUGT2 is adjacent to GmUGT1 and GmUGT7 which are able to glucosylate isoflavones at *O*-7 position and flavones at *O*-4′ position (Funaki et al., 2015). PlUGT2 was then expected to be a likely candidate for our purposes in this study. Heterologous expression experiments clearly showed that PlUGT2 not only catalyzes a 4′-*O-*glucosylation of isoflavones (daidzein and genistein) but also glucosylates daidzein and genistein at *O*-7 position (**Figure 4**). The dual biochemical activities may suggest that the acceptor-binding pocket of PlUGT2 would be much longer and larger than daidzein or genistein, allowing them to be easily positioned in two different directions. The multiple activities of a UGT toward an acceptor at different hydroxyl groups were also previously observed for the *Medicago truncatula* UGT with quercetin (Shao et al., 2005). The structural basis for the dual functionality of PlUGT2 could be further resolved by docking its structural model with the acceptors as well as the donor UDP-glucose. Interestingly, when daidzein or genistein was used as the substrate, PlUGT2 catalyzed a sequential glucosylation, converting them to 4′- or 7-*O-*mono-glucosides and then the mono-glucosides to the 4′,7-*O-*diglucosides (**Figure 4**). It should be mentioned that although daidzein or genistein was efficiently glucosylated by PlUGT2 at *O*-4′ or *O*-7 position, its activities toward daidzin (7-*O-*glucose-daidzein), sophoricoside (4′-*O-*glucose-daidzein), puerarin (8-*C-*glucose-daidzein), and genistin (7-*O-*glucose-genistein) were pretty low (**Table 1**), suggesting that a glucosyl residue attached to daidzein or geinstein might form steric hindrance for the secondary glucosylation by PlUGT2. On the other hand, PlUGT2 efficiently catalyzes a 7-*O-*glucosylation with formononetin (4′-methoxyl-daidzein) yielding ononin (**Table 1**, Supplementary Figure S4F), which indicated that the methyl group attachment does not interfere with the glucosylation. In addition, despite the closer relationship of PlUGT18 with UGT74W1 (**Figure 3**), PlUGT18 did not show the activity as does by UGT74W1 which specifically glucosylates genistein at *O*-4′ position and was not active with any substrates used in our previous study (Li et al., 2014; Ruby et al., 2014), providing another example that the biochemical function of UGTs could not be predicted only by their primary sequences.

Due to the lack of genetic transformation system for non-model plant species, a combination of enzyme biochemical property *in vitro*, gene expression and metabolite accumulation *in vivo* has been used in many cases to deduce the *in vivo* biochemical roles of plant secondary metabolism enzymes. In *P. lobata*, isoflavonoid *O-*glucosides mostly accumulate in the roots (**Figure 2**). Among these *O-*glucosides, 7-*O-*glucosides with free 4′-hydroxyl group such as daidzin and genistin are predominant while 4′-*O-*glucosides such as sophoricoside, daidzein 4′-*O-*glucoside, daidzein 4′,7-*O-*diglucoside, and puerarin 4′-*O-*glucoside accumulate at extremely low levels (He et al., 2011; Yu et al., 2011; Zhang et al., 2013), which was also clearly seen in the present work (**Figure 2**). This accumulation pattern suggested that the 7-*O-*glucosylation activity must be far more prevalent than 4′-*O-*glucosylation activity in *P. lobata*. In fact, enzymes catalyzing the 7-*O-*glucosylation including PlUGT1 and PlUGT13 (Li et al., 2014), GT04F14 (He et al., 2011), and PlUGT2 reported in this study, have been identified from *P. lobata*, whereas PlUGT2 is the only enzyme catalyzing the 4′-*O-*glucosylation identified from this plant so far. It is not clear whether these 7-*O-*PlUGTs (PlUGT1, PlUGT13, GT04F14, and PlUGT2) all together contribute to the 7-*O-*glucosylation *in vivo*, but based on the combination of enzyme substrate specificity and the accumulation pattern of both gene transcript abundance and metabolite profiling, PlUGT1 was suggested to be the enzyme responsible for the 7-*O-*glucosylation in *P. lobata* (Li et al., 2014). Consistent with the prevalence of 7-*O-*glucosides and the low accumulation of 4′-*O-*glucosides, enzyme assays for daidzein or geinstein 7-*O-*glucosylation by PlUGT1 showed much higher activities (1.1 ± 0.04 s^-1^ for genistein, 1.1 ± 0.03 s^-1^ for daidzein) than those by PlUGT2 (0.176 ± 0.005 s^-1^ for genistein, 0.068 ± 0.003 s^-1^ for daidzein, **Table 1**). It should be noted that the reaction rates mentioned here were calculated by the consumption of substrates in the *in vitro* enzyme assays. The 7-*O-*glucosides are the sole reaction products by PlUGT1 toward daidzein or genistein while multiple glucosides including 7-*O-* and 4′-*O-*mono-glucosides, and 4′,7-*O-*diglucosides are produced by PlUGT2 (**Figure 4**). Therefore, the activity of PlUGT2 specific for the 4′-*O-*glucosylation would be more than 10-fold lower than that of PlUGT1 for the 7-*O-*glucosylation. The low reaction rate of PlUGT2 for the 4′-*O-*glucosylation *in vitro* is consistent with the low accumulation of isoflavonoid 4′-*O-*glucosides in *P. lobata*. *P. lobata* isoflavonoid 4′-*O-*glucosides were majorly detected in its roots relative to other organs (**Figure 2**), which also matches the accumulation pattern of *PlUGT2* transcripts (**Figure 5**). PlUGT2 was placed in the subgroup B clade with 7-*O-*UGTs in our constructed phylogenetic tree (**Figure 3**). UGT members within the subgroup B family usually have broad substrate specificities (Funaki et al., 2015). However, *in vitro* enzyme assays demonstrated that PlUGT2 seems to have substrate preference for isoflavones other than flavones (**Table 1**). Thus, PlUGT2 likely has been recruited from 7-*O-*UGT activity to 4′-*O-*UGT activity specific for isoflavones.

## Conclusion

Although, the physiological role of PlUGT2 in *P. lobata* is not clear, the identification of PlUGT2 may provide a useful enzyme catalyst for an efficient biotransformation of isoflavones and other natural products for food or pharmacological purposes. For instance, in this case, PlUGT2 is capable of glucosylating puerarin which has been reported to exhibit great pharmacological activities but is limited for clinical trials due to its low water solubility. The report of PlUGT2 here would provide such an opportunity; at least provide an enzyme template for designing novel enzyme catalysts, for increasing puerarin water solubility and in turn pushing forward for its applications.

## Author Contributions

YZ designed this study; XW performed the gene cloning and biochemical reactions; RF performed the protein expression in *E. coli*; JL provided the assistance in the *in vitro* reactions; CL provided the assistance in LC–MS or HPLC analysis; XW and YZ wrote the manuscript.

## Conflict of Interest Statement

The authors declare that the research was conducted in the absence of any commercial or financial relationships that could be construed as a potential conflict of interest.

## References

[B1] Cheynier V. Comte G. Davies K. M. Lattanzio V. Martens S. (2013). Plant phenolics: recent advances on their biosynthesis, genetics, and ecophysiology. *Plant Physiol. Biochem.* 72 1–20. 10.1016/j.plaphy.2013.05.009 23774057

[B2] Deavours B. E. Liu C. J. Naoumkina M. A. Tang Y. Farag M. A. Sumner L. W. (2006). Functional analysis of members of the isoflavone and isoflavanone O-methyltransferase enzyme families from the model legume *Medicago truncatula*. *Plant Mol. Biol.* 62 715–733. 10.1007/s11103-006-9050-x 17001495 PMC2862459

[B3] Du G. Zhao H. Song Y. Zhang Q. Wang Y. (2011). Rapid simultaneous determination of isoflavones in *Radix puerariae* using high-performance liquid chromatography-triple quadrupole mass spectrometry with novel shell-type column. *J. Sep. Sci.* 34 2576–2585. 10.1002/jssc.201100295 21898802

[B4] Fang C. Wan X. Tan H. Jiang C. (2006a). Identification of isoflavonoids in several kudzu samples by high-performance liquid chromatography coupled with electrospray ionization tandem mass spectrometry. *J. Chromatogr. Sci.* 44 57–63. 10.1093/chromsci/44.2.57 16620495

[B5] Fang C. Wan X. Tan H. Jiang C. (2006b). Separation and determination of isoflavonoids in several kudzu samples by high-performance capillary electrophoresis (HPCE). *Ann. Chim.* 96 117–124. 10.1002/adic.200690002 16734027

[B6] Ferreyra M. L. F. Rius S. P. Casati P. (2012). Flavonoids: biosynthesis, biological functions, and biotechnological applications. *Front. Plant Sci.* 3:222 10.3389/fpls.2012.00222 PMC3460232 23060891

[B7] Funaki A. Waki T. Noguchi A. Kawai Y. Yamashita S. Takahashi S. (2015). Identification of a highly specific isoflavone 7-O-glucosyltransferase in the soybean (*Glycine max* (L.) Merr.). *Plant Cell Physiol.* 56 1512–1520. 10.1093/pcp/pcv072 26019269

[B8] Gachon C. M. Langlois-Meurinne M. Saindrenan P. (2005). Plant secondary metabolism glycosyltransferases: the emerging functional analysis. *Trends Plant Sci.* 10 542–549. 10.1016/j.tplants.2005.09.007 16214386

[B9] Hansen S. F. Harholt J. Oikawa A. Scheller H. V. (2012). Plant glycosyltransferases beyond CAZy: a perspective on DUF families. *Front. Plant Sci.* 3:59 10.3389/fpls.2012.00059 PMC3355507 22629278

[B10] He X. Z. Blount J. W. Ge S. J. Tang Y. H. Dixon R. A. (2011). A genomic approach to isoflavone biosynthesis in kudzu (*Pueraria lobata*). *Planta* 233 843–855. 10.1007/s00425-010-1344-1 21221632

[B11] Jiang J. R. Yuan S. Ding J. F. Zhu S. C. Xu H. D. Chen T. (2008). Conversion of puerarin into its 7-O-glycoside derivatives by *Microbacterium oxydans* (CGMCC 1788) to improve its water solubility and pharmacokinetic properties. *Appl. Microbiol. Biotechnol.* 81 647–657. 10.1007/s00253-008-1683-z 18795283

[B12] Kayano S. Matsumura Y. Kitagawa Y. Kobayashi M. Nagayama A. Kawabata N. (2012). Isoflavone C-glycosides isolated from the root of kudzu (*Pueraria lobata*) and their estrogenic activities. *Food Chem.* 134 282–287. 10.1016/j.foodchem.2012.02.137

[B13] Keung W. M. Lazo O. Kunze L. Vallee B. L. (1996). Potentiation of the bioavailability of daidzin by an extract of *Radix puerariae*. *Proc. Natl. Acad. Sci. U.S.A.* 93 4284–4288. 10.1073/pnas.93.9.4284 8633056 PMC39527

[B14] Kinjo J. E. Furusawa J. I. Baba J. Takeshita T. Yamasaki M. Nohara T. (1987). Studies on the constituents of *Pueraria lobata*. III. Isoflavonoids and related compounds in the roots and the voluble stems. *Chem. Pharm. Bull.* 35 4846–4850. 10.1248/cpb.35.4846

[B15] Li D. Park S. H. Shim J. H. Lee H. S. Tang S. Y. Park C. S. (2004). In vitro enzymatic modification of puerarin to puerarin glycosides by maltogenic amylase. *Carbohydr. Res.* 339 2789–2797. 10.1016/j.carres.2004.09.017 15542087

[B16] Li J. Li Z. B. Li C. F. Gou J. B. Zhang Y. S. (2014). Molecular cloning and characterization of an isoflavone 7-O-glucosyltransferase from *Pueraria lobata*. *Plant Cell Rep.* 33 1173–1185. 10.1007/s00299-014-1606-7 24700248

[B17] Modolo L. V. Blount J. W. Achnine L. Naoumkina M. A. Wang X. Dixon R. A. (2007). A functional genomics approach to (iso)flavonoid glycosylation in the model legume *Medicago truncatula*. *Plant Mol. Biol.* 64 499–518. 10.1007/s11103-007-9167-6 17437063

[B18] Ohshima Y. Okuyama T. Takahashi K. Takizawa T. Shibata S. (1988). Isolation and high performance liquid chromatography (HPLC) of isoflavonoids from the *Pueraria* root. *Planta Med.* 54 250–254. 10.1055/s-2006-962420 3174862

[B19] Prasain J. K. Reppert A. Jones K. Moore D. R. Barnes S. Lila M. A. (2007). Identification of isoflavone glycosides in *Pueraria lobata* cultures by tandem mass spectrometry. *Phytochem. Anal.* 18 50–59. 10.1002/pca.951 17260698

[B20] Rong H. Stevens J. F. Deinzer M. L. Cooman L. D. Keukeleire D. D. (1998). Identification of isoflavones in the roots of *Pueraria lobata*. *Planta Med.* 64 620–627. 10.1055/s-2006-957534 17253303

[B21] Ruby Kumar R. J. S. Vishwakarma R. K. Singh S. Khan B. M. (2014). Molecular cloning and characterization of genistein 4′-O-glucoside specific glycosyltransferase from *Bacopa monniera*. *Mol. Biol. Rep.* 41 4675–4688. 10.1007/s11033-014-3338-8 24664316

[B22] Saito K. Yonekura-Sakakibara K. Nakabayashi R. Higashi Y. Yamazaki M. Tohge T. (2013). The flavonoid biosynthetic pathway in *Arabidopsis*: structural and genetic diversity. *Plant Physiol. Biochem.* 72 21–34. 10.1016/j.plaphy.2013.02.001 23473981

[B23] Shao H. He X. Achine L. Blount J. Dixon R. Wang X. (2005). Crystal structures of a multifunctional triterpene/flavonoid glycosyltransferase from *Medicago truncatula*. *Plant Cell* 17 3141–3154. 10.1105/tpc.105.035055 16214900 PMC1276034

[B24] Shi J. Chang Q. Shen L. Chen D. (2006). Chemical constituents from *Pueraria lobata*. *J. Chinese Pharm. Sci.* 15 248–250.

[B25] Song W. Li Y. J. Qiao X. Qian Y. Ye M. (2014). Chemistry of the Chinese herbal medicine Puerariae Radix (Ge-Gen): a review. *J. Chinese Pharm. Sci.* 23 347–360.

[B26] Tang X. L. Liu X. J. Tian Q. Zhang W. (2012). Dynamic oxidative stress and DNA damage induced by oestrogen deficiency and protective effects of puerarin and 17 beta-oestradiol in ovariectomized rats. *Basic Clin. Pharmacol.* 111 87–91. 10.1111/j.1742-7843.2012.00864.x 22333267

[B27] Veitch N. C. (2009). Isoflavonoids of the Leguminosae. *Nat. Prog. Rep.* 26 776–802. 10.1039/b616809b 19471685

[B28] Vogt T. Jones P. (2000). Glycosyltransferases in plant natural product synthesis: characterization of a supergene family. *Trends Plant Sci.* 5 380–386. 10.1016/S1360-1385(00)01720-9 10973093

[B29] Wang X. Li S. T. Li J. Li C. F. Zhang Y. S. (2015). De novo transcriptome sequencing in *Pueraria lobata* to identify putative genes involved in isoflavones biosynthesis. *Plant Cell Rep.* 34 733–743. 10.1007/s00299-014-1733-1 25547742

[B30] Wong K. H. Li G. Q. Li K. M. Razmovski-Naumovski V. Chan K. (2011). Kudzu root: traditional uses and potential medicinal benefits in diabetes and cardiovascular diseases. *J. Ethnopharmacol.* 134 584–607. 10.1016/j.jep.2011.02.001 21315814

[B31] Wong K. H. Razmovski-Naumovski V. Li K. M. Li G. Q. Chan K. (2015). Comparing morphological, chemical and anti-diabetic characteristics of Puerariae Lobatae Radix and Puerariae Thomsonii Radix. *J. Ethnopharmacol.* 164 53–63. 10.1016/j.jep.2014.12.050 25560667

[B32] Xiao J. B. Muzashvili T. S. Georgiev M. I. (2014). Advances in the biotechnological glycosylation of valuable flavonoids. *Biotechnol. Adv.* 32 1145–1156. 10.1016/j.biotechadv.2014.04.006 24780153

[B33] Yasuda T. Endo M. Kon-no T. Kato T. Mitsuzuka M. Ohsawa K. (2005). Antipyretic, analgesic and muscle relaxant activities of pueraria isoflavonoids and their metabolites from *Pueraria lobata* Ohwi-a traditional Chinese drug. *Biol. Pharm. Bull.* 28 1224–1228. 10.1248/bpb.28.1224 15997103

[B34] Yonekura-Sakakibara K. Hanada K. (2011). An evolutionary view of functional diversity in family 1 glycosyltransferases. *Plant J.* 66 182–193. 10.1111/j.1365-313X.2011.04493.x 21443631

[B35] Yu Y. L. Liao Y. T. Li X. A. Ye Y. Ke C. Q. Li X. Q. (2011). Isoflavonoid glycosides from the flowers of *Pueraria lobata*. *J. Asian Nat. Prod. Res.* 13 284–289. 10.1080/10286020.2011.554404 21462030

[B36] Zhang J. Guo W. S. Tian B. X. Sun M. H. Li H. Zhou L. N. (2015). Puerarin attenuates cognitive dysfunction and oxidative stress in vascular dementia rats induced by chronic ischemia. *Int. J. Clin. Exp. Pathol.* 8 4695–4704. 26191159 PMC4503031

[B37] Zhang Z. Lam T.-N. Zuo Z. (2013). *Radix puerariae*: an overview of its chemistry, pharmacology, pharmacokinetics, and clinical use. *J. Clin. Pharmacol.* 53 787–811. 10.1002/jcph.96 23677886

