# Supplementary material for: Molecular Cloning and Functional Characterization of a Novel (Iso)flavone 4′,7-O-diglucoside Glucosyltransferase from Pueraria lobata
Source: Front Plant Sci. 2016 Mar 31;7:387. doi: 10.3389/fpls.2016.00387 (PMC4814453; doi:10.3389/fpls.2016.00387)
Supplement: Supplementary file 3 [file Image_1.PDF]

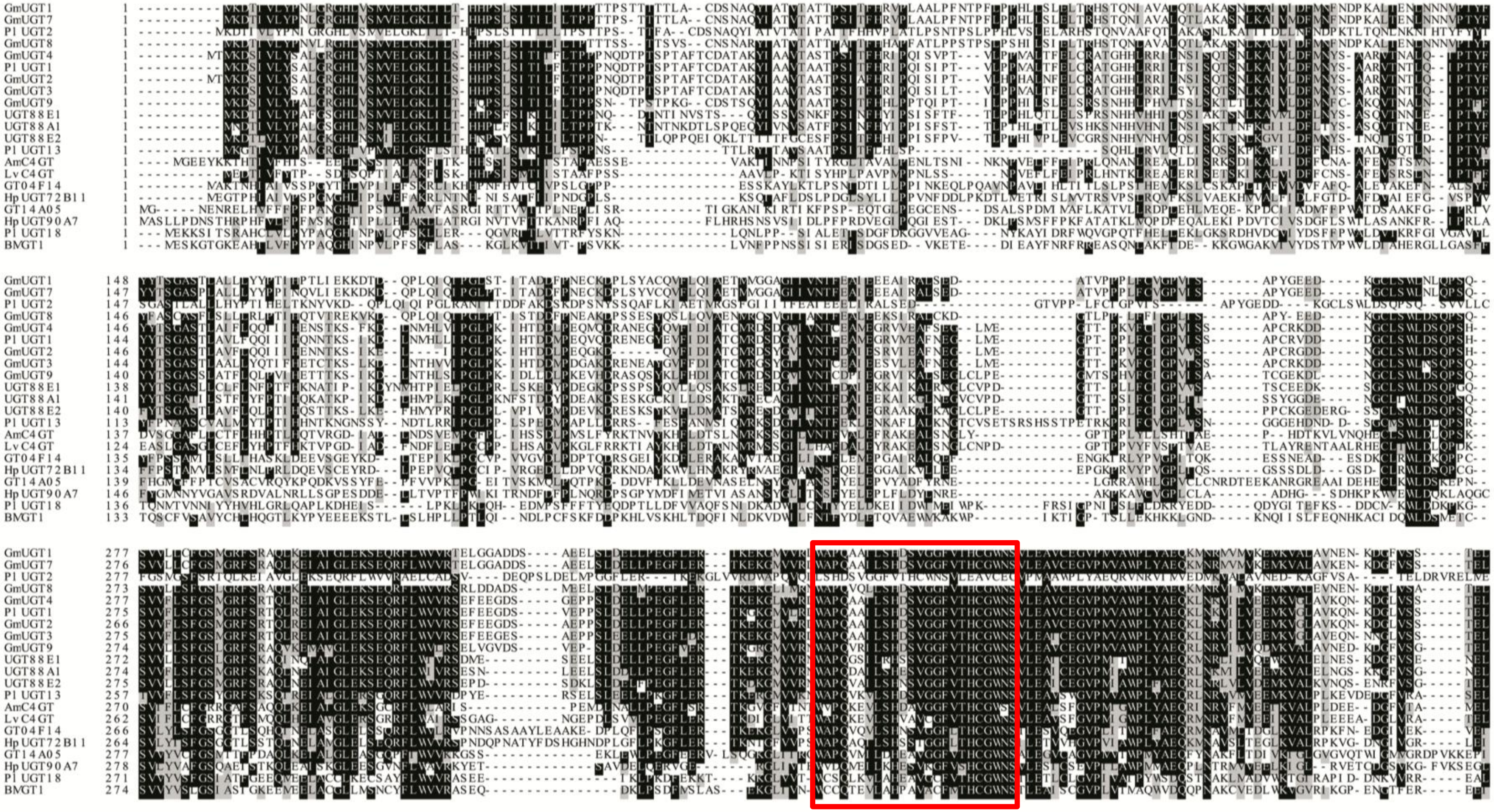

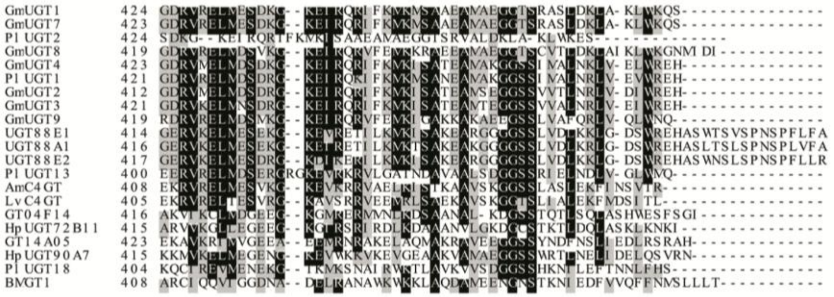

Supplementary Figure 1. Multiple alignment of deduced amino acid sequences of PlUGT2 with other representative UGTs. Black shaded and gray shaded boxes show identical and similar amino acids, respectively. The conserved PSPG motif (potential UDP-binding domain) at the C-termini is indicated by the red box. The UGTs names and accession numbers are shown in the Figure 3 legend.

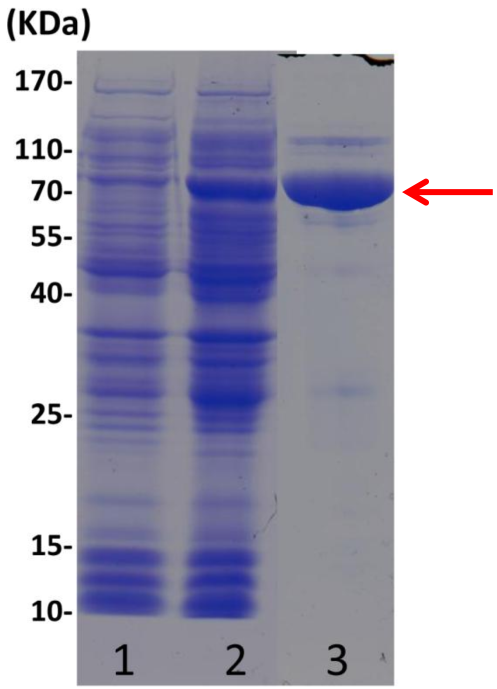

Supplementary Figure 2. SDS-PAGE examination of the expression and purification of recombinant PlUGT2 protein. 1, Crude extract of *E. coli* cells harboring pGEM-2T-*PlUGT2* before induction; 2, Soluble proteins of *E. coli* cells harboring pGEM-2T-*PlUGT2* induced by IPTG for 16 h; (3) the recombinant OsGPX5 protein purified with Glutathione Sepharose 4B (GE Healthcare) and desalted by 30 kDa ultra centrifugal filter (Millipore). Protein band of PlUGT2 is indicated by red arrowhead.

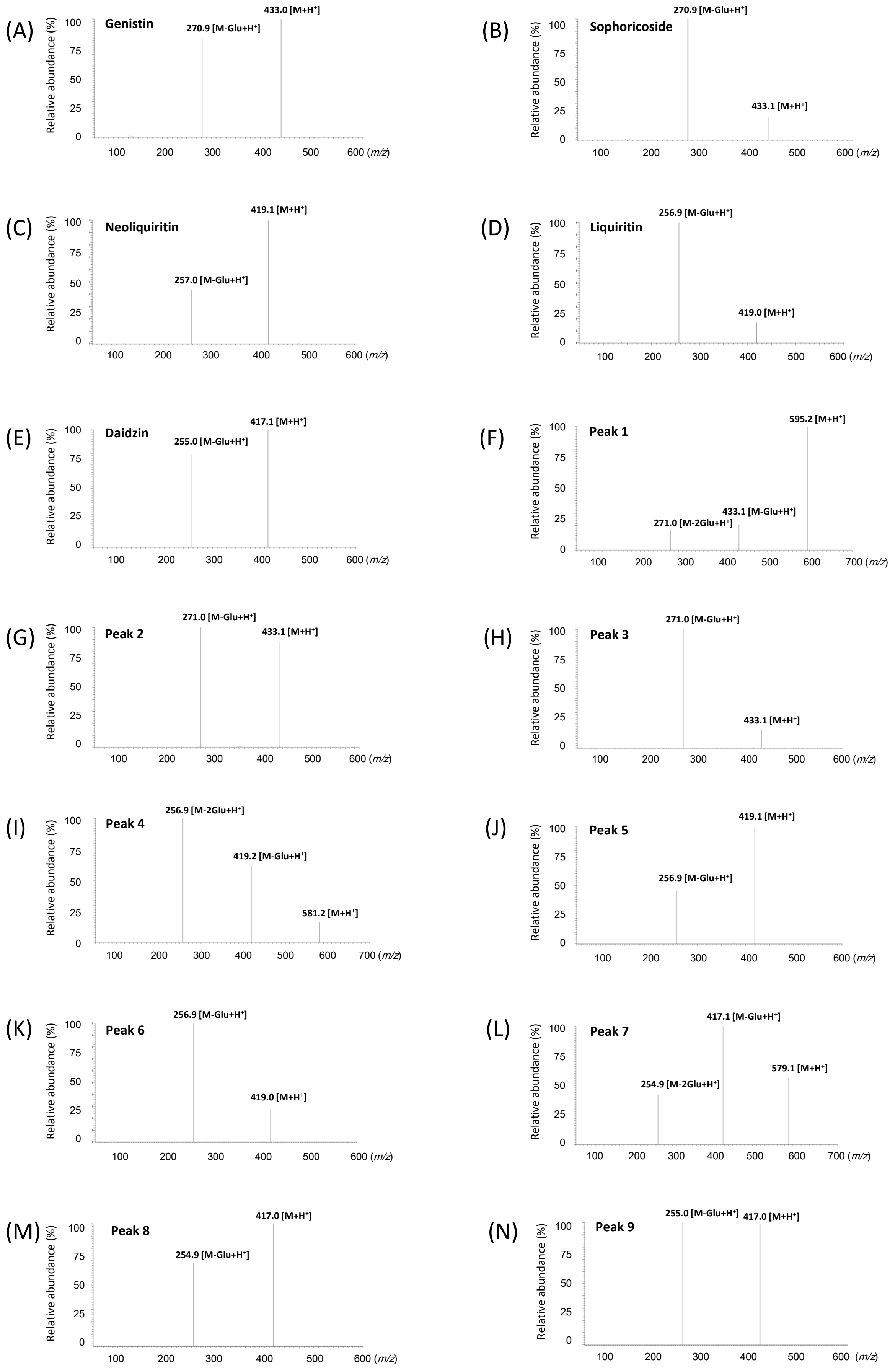

Supplementary Figure 3 (continuous)

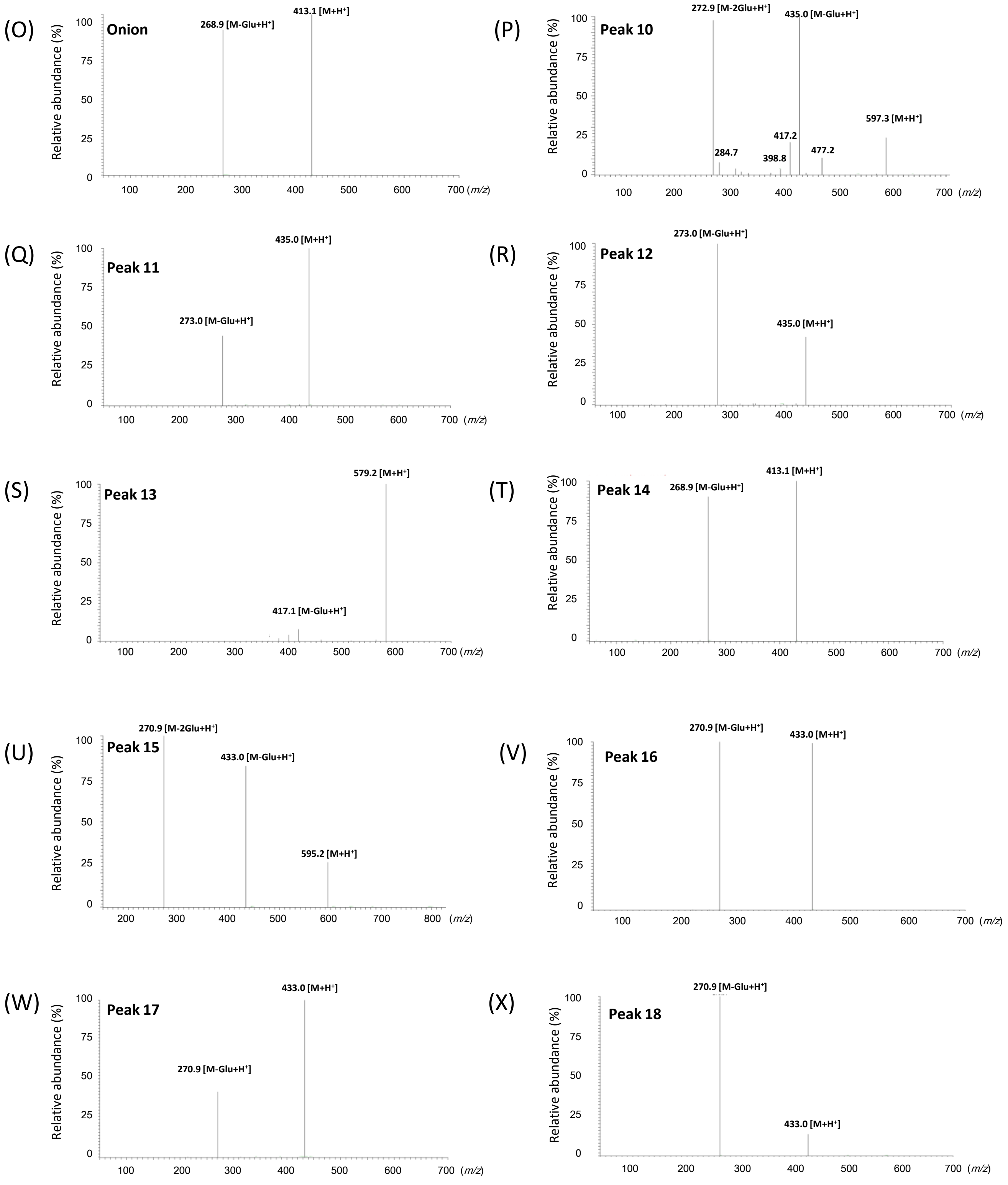

Supplementary Figure 3

Supplementary Figure 3. Mass spectra of the products (peaks1−18) formed by the in vitro assays of PlUGT2 with various (iso)flavone substrates. (A)−(E) and (O) are mass spectra of the authentic standards. (A), genistin (genistein 7-*O*-glucoside); (B), sophoricoside (genistein 4'-*O*-glucoside); (C), neoliquiritin (liquiritigenin 7-*O*-glucoside); (D), liquiritin (liquiritigenin 4'-*O*-glucoside); (E), daidzin (daidzein 7-*O*-glucoside); (O), ononin (formononetin 7-*O*-glucoside). (F)−(H) showed the mass spectra of the products that PlUGT2 reacted with genistein; (I)−(K) showed the mass spectra of the products that PlUGT2 reacted with liquiritigenin; (L)−(N) showed the mass spectra of the products that PlUGT2 reacted with daidzein; (P)−(R) showed the mass spectra of the products that PlUGT2 reacted with naringenin; (S)−(T) showed the mass spectra of the products that PlUGT2 reacted with purearin or formononetin repectively; (U)−(X) showed the mass spectra of the products that PlUGT2 reacted with 3',4',7-trihydroxyisoflavone. The chemical structures of the products (peak1−14) are shown in Figure 1.

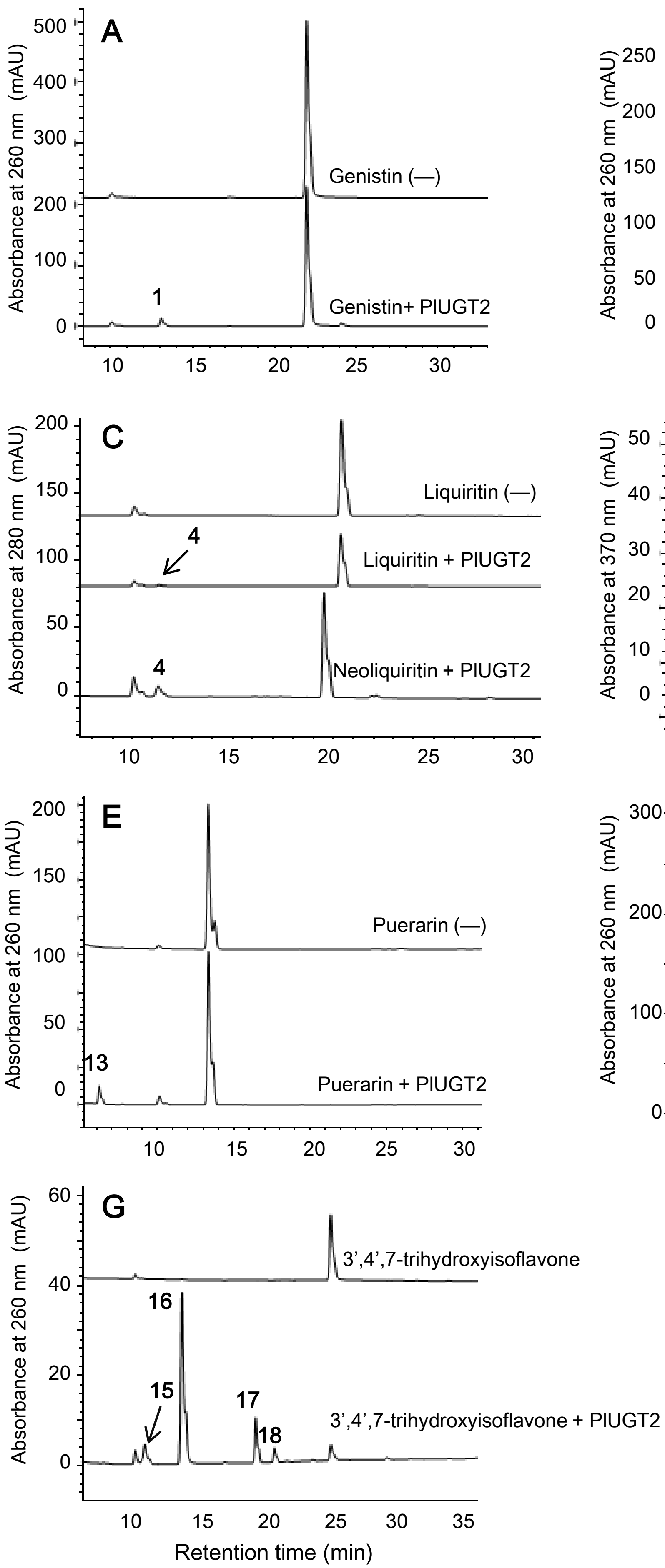

Supplementary Figure 4

Supplementary Figure 4. HPLC analysis of the products extracted from the *in vitro* reactions of PlUGT2 with other (iso)flavone acceptors. genistin (A), sophoricoside (B), liquiritin or neoliquiritin (C), naringenin (D), purearin (E), formononetin (F), and 3',4',7-trihydroxyisoflavone (G). (−) indicates control reactions without the addition of PlUGT2. Peak 1, genitein 4',7-*O*-diglucoside; peak 4, liquiritigenin 4',7-*O*-diglucoside; peak 10−12 are naringenin 4',7-*O*-diglucoside, naringenin 7-*O*-glucoside, and naringenin 4'-*O*-glucoside respectively;. peak 13, purearin mono-glucoside; peak14, ononin (formononetin 7-*O*-glucoside); peak 15 is likely to be diglucoside of 3',4',7-trihydroxyisoflavone, but the glusosylation positions are not clear, while peak 16−18 are mono-glucosides of 3',4',7-trihydroxyisoflavone, which might be attached a single sugar to the hydroxyl groups at either C'-3, C'-4 or C-7 positions. The mass spectra of the reaction products (peak 1, 4, 10−18) were shown in Fig. S3.
